# Cause of death coding in asthma

**DOI:** 10.1186/s12874-024-02238-x

**Published:** 2024-06-05

**Authors:** Alexandria Chung, George Addo Opoku-Pare, Holly Tibble

**Affiliations:** 1 Usher Institute, University of Edinburgh, Edinburgh, Scotland; 2 Asthma UK Centre for Applied Research, Edinburgh, Scotland

**Keywords:** ICD-10, Mortality, Asthma

## Abstract

**Background:**

While clinical coding is intended to be an objective and standardized practice, it is important to recognize that it is not entirely the case. The clinical and bureaucratic practices from event of death to a case being entered into a research dataset are important context for analysing and interpreting this data. Variation in practices can influence the accuracy of the final coded record in two different stages: the reporting of the death certificate, and the International Classification of Diseases (Version 10; ICD-10) coding of that certificate.

**Methods:**

This study investigated 91,022 deaths recorded in the Scottish Asthma Learning Healthcare System dataset between 2000 and 2017. Asthma-related deaths were identified by the presence of any of ICD-10 codes J45 or J46, in any position. These codes were categorized either as relating to asthma attacks specifically (status asthmatic; J46) or generally to asthma diagnosis (J45).

**Results:**

We found that one in every 200 deaths in this were coded as being asthma related. Less than 1% of asthma-related mortality records used both J45 and J46 ICD-10 codes as causes. Infection (predominantly pneumonia) was more commonly reported as a contributing cause of death when J45 was the primary coded cause, compared to J46, which specifically denotes asthma attacks.

**Conclusion:**

Further inspection of patient history can be essential to validate deaths recorded as caused by asthma, and to identify potentially mis-recorded non-asthma deaths, particularly in those with complex comorbidities.

## Background

In countries with full civil registration coverage, death records are used to legally certify someone as deceased, to monitor mortality patterns and the epidemiology of specific conditions, to inform family members about their relative’s medical history which may be of relevance to themselves, and to act as a form of emotional closure for the family and healthcare team who had looked after the patient [1]. The correct and accurate recording of death is therefore important on a personal, national, and international level and this information is processed by these different stakeholders in a variety of ways.

The Medical Certificate of Cause of Death (MCCD) [1] is an internationally standardized form which requires a qualified medical practitioner to complete. In the UK, the issuing of a MCCD is the legal responsibility of the doctor who had attended to the patient during their last illness within the past 28 days [2]. In practical terms, this tends to fall upon the patient’s most recent hospital consultant or General Practitioner who is expected to assign the final cause of death except in the rare circumstance where certification is conducted by a coroner. Cause of death assignment is often based on the patient’s medical records, relevant investigation results, and the responsible doctor’s experience of meeting and caring for the patient [2].

Legally, deaths must be registered within 5 days unless referred to a coroner whom may also initiate a post-mortem or an inquest [2]. In such cases, the coroner then shares their findings with the registrar, and their final diagnosis is utilized instead of the information provided on MCCD for the purpose of officially registering the death [3]. The MCCD assignment is divided into two parts: the causes of death (Part I) and any other health or circumstantial condition which is thought to have indirectly significantly contributed to a person’s final cause of death (Part II) [1]. Part I is further divided into the immediate cause of death (Part Ia) and the contributing causes (if required; Part Ib) which sequentially led to this event such that the diagnosis on the lowest line caused the conditions above it (thus known as the Underlying Cause Of Death, or UCOD).

The reported causes of death are then coded according to the International Classification of Diseases (ICD) by a trained coder or validated clinical coding software. This is conducted by the Office for National Statistics (ONS) in England and Wales and National Records of Scotland (NRS) in Scotland. Between 2000 and 2017, NRS used the automated Mortality Medical Data System (MMDS) for cause of death coding. Since then, the NRS transitioned to Iris, as recommended by the World Health Organization (WHO) [4], but the ONS have been using version 5.8 of the Multicausal and Unicausal Selection Engine, or MUSE, since January 2022 [5].

**Fig. 1:**
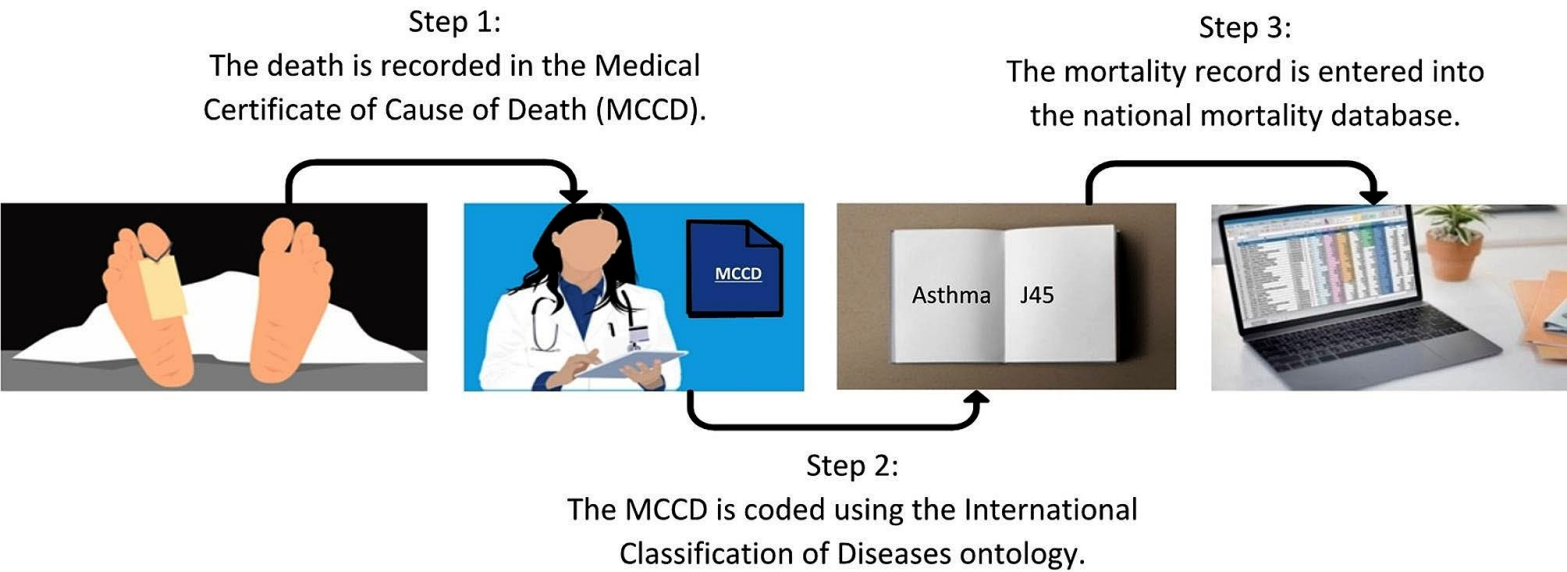
The chain of events from death to record being entered in the national mortality database

While clinical coding is intended to be an objective and standardized practice, it is important to recognize that it is not entirely devoid of bias. Sometimes, the available medical information may be ambiguous or incomplete, making it challenging to determine the exact cause of death [6]. In such cases, clinical coders must rely on their judgment and interpretation to assign the most appropriate code. Additionally, coding guidelines evolve over time to accommodate new medical knowledge and practices [7]. Changes in guidelines or updates may require coders to adapt their coding practices, which can introduce inconsistencies until widespread adoption and understanding of the new guidelines are achieved [8, 9]. This bias can introduce variability in the coding process, leading to discrepancies, particularly in assigning codes for complex or rare conditions [10]. As well as ambiguity and uncertainty, there can be cultural and political influences on how cause of death is reported and subsequently coded (Fig. 1). Suicide is notoriously poorly reported, due in part to the sensitivity of the event and the legality of suicidal behavior, resulting in substantial under-reporting [11, 12].

Shifts and variations in the use of the specific reporting and coding practices have two primary impacts in public health research. Firstly, comparisons between different populations may be hindered due to differences (either due to administrative or cultural variations), resulting in poor inference about associations between population-level factors and mortality. Secondly, it also hinders within population estimates over a duration of time. For example, interrupted time-series analyses may be affected by changes which do not reflect the underlying mortality rate. Additionally, the performance of risk prediction models may become biased by these differences for testing done by a temporal split if the practices have changed [13–15].

In this study, we aimed to investigate how asthma deaths in Scotland are coded, and whether there has been any change in practices over time.

## Methods

### Mortality data

In UK population mortality research datasets, the core variables are the personal identifier (typically pseudonymized), the date of death, and the causes of death. Additional variables may include the location of death, date of death registration, and details of coronial enquiry [16, 17].

The causes of death are presented as the ‘primary’ cause (the UCOD), and up to ten secondary causes, ordered from 0 to 9 (as per the sequence of events recorded in the first part of the MCCD). There is also a field denoting the version of the ICD that was in use in the data. In the ICD-10 ontology, each code is of four to six characters in length: the first three characters indicate the category of diagnosis, and characters four to six indicate aetiology, anatomical site, severity, or other clinical details [18].

### The asthma learning healthcare system study population

The Asthma Learning Healthcare System (ALHS) study recruited over half a million patients from 75 general practices in Scotland, with primary care records linked to national accident and emergency (A&E), hospital, and mortality datasets, for all participants [19]. The study period was between January 2000 and March 2017. In this dataset, each diagnostic code is formatted to be 5 characters in length, with the form of a letter (denoting the chapter) plus either two or three digits (and one or two trailing spaces) as per ICD-10.

### Analysis plan

Mortality records were reviewed to identify duplicated codes in different positions (primary and secondary). The number of (de-duplicated) codes recorded per death and change over time (by year) were examined, as well as the odds ratios of code incidence compared to those without asthma as a primary or contributing cause of death.

Asthma-related deaths were identified by the presence of any of J45 or J46, in any position. These codes were categorized either as relating to asthma attacks specifically (status asthmatic; J46) or generally to asthma diagnosis (J45). Our study did not use J44 codes, which incorporates chronic obstructive asthmatic bronchitis under J448, to identify cases as this category pertains primarily to chronic obstructive pulmonary disease (COPD) rather than asthma. In individuals who died of asthma-related causes, the location (primary versus secondary) of the code(s) were assessed, and specifically whether they were more commonly asthma or asthma-attack codes.

Any counts of five or below have been suppressed to protect patient confidentiality. Analysis was conducted in the R programming language (version 4.3.1), and code is available on the open-source platform GitHub at https://github.com/hollytibble/asthma_mortality_ICD.

## Results

**Fig. 2:**
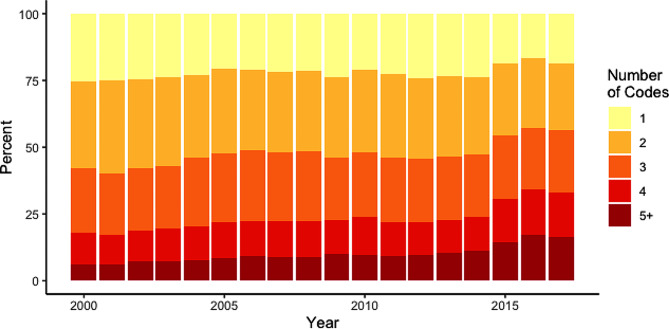
Number of cause of death codes provided per death, per year

The ALHS dataset contained records for 91,022 deaths (excluding stillbirths) between January 1st 2000, and March 31st, 2017. After duplicate removal, there were a median of 2 causes coded per death (interquartile range 2 to 3). The number of codes increased on average over time, with 25.4% having only a single code, and 6.0% having five or more in 2000, compared to 18.6% with a single code and 16.3% with five or more in 2017 (Fig. 2).

There were 487 deaths in this time (0.54%) which included asthma-related causes. There was no observed change in frequency by year. Fewer than 5 of these deaths had both J45 and J46 codes (in any position; less than 1%), and 92% (450/487) deaths had only the J45 code, rather than the J46 code.

For the deaths mentioning asthma as a cause, it was the primary cause of death in 190 cases (39.0%). Of these, the most common code was J45 (82.1%, *n* = 156). Across years, the median percentage of primary asthma codes that were J45, rather than J46, was 87.1% (interquartile range of annual percentages = 68.2 to 89.3%), with no consistent trend over time.

There was an infection reported as a contributing or primary cause of death for 56.4% of deaths with asthma (J45) as the primary cause, ≤ 14.7% (*n* ≤ 5, exact number masked to protect patient confidentiality) when asthma attack (J46) was the primary cause. In comparison, infection was reported as a contributing or primary cause of death for 24.2% of deaths in which asthma was only a secondary cause (*n* = 72/297), and 25.2% (*n* = 22,793/90,535) of deaths in which asthma was not recorded in any position in the death record. Compared to ‘no asthma’, J45 as primary cause had 3.85 times higher odds of recorded infection (95% CI = 2.80 to 5.28), while there was no significant difference for J46 primary cause or non-primary asthma cause. The difference in infection record between those with J45 and J46 as the primary cause was also significant (7.51 times higher odds with J45 as primary code, 95% CI = 2.76 to 20.41).

When the primary cause of death was asthma-related, the most common secondary cause (any position) was unspecified bronchopneumonia (26.3%; top five presented in Table 1).

**Table 1:** Top five most common secondary causes of death, for deaths with asthma-related primary cause

Secondary Cause of Death	ICD10 Code	*N* (%)
Unspecified bronchopneumonia	J180	50 (16.3%)
Unspecified pneumonia	J189	28 (14.7%)
Unspecified chronic ischemic heart disease	I259	17 (8.9%)
Other chronic obstructive pulmonary disease*	J448	11 (5.8%)
Dementia	F03	9 (4.7%)

*Note* including asthma-COPD overlap

However, given the general prevalence of some of these as secondary causes, the odds of unspecified chronic ischemic heart disease was in fact lower when asthma was the primary cause of death than when asthma was not recorded as a cause at all (odds ratio = 0.56, 95% CI = 0.34 to 0.93). There were four secondary causes with odds ratios of higher than 3, as listed in Table 2.

**Table 2:** Secondary causes of death with Odds Ratio of greater than 3 of presence, for deaths with asthma-related primary cause compared to non-asthma-related deaths

Secondary Cause of Death	ICD10 Code	Odds Ratio (95% Confidence Interval)
Other chronic obstructive pulmonary *	J448	28.62 (15.31 to 53.46)
Respiratory arrest	R092	12.80 (5.62 to 29.17)
Other secondary pulmonary hypertension	I272	6.66 (2.72 to 16.28)
Respiratory failure, unspecified	J969	3.9 (1.92 to 7.95)

*Note* Including asthma-COPD overlap

For the 297 deaths with asthma as a secondary cause rather than the primary cause, the most common primary cause chapter was diseases of the circulatory system (ICD chapter I; 42.1%). The top five chapters by prevalence are shown in Table 3.

**Table 3:** Primary causes of death by ICD10 Chapter, for deaths with asthma recorded as a secondary cause of death

Primary ICD Chapter Name (Code)	Percent
Circulatory system (I)	42.1%
Neoplasms (C)	21.2%
Respiratory system (J)	9.1%
Digestive system (K)	6.1%
Nervous System (G)	5.1%

There were 1.93 times higher odds of endocrine, nutritional and metabolic diseases (chapter E) when asthma was a secondary cause (compared to asthma not recorded in any position, 95% CI 1.03 to 3.63), 1.59 times higher odds for diseases of the circulatory system (chapter I, 95% CI = 1.26 to 2.00), but an odds ratio of 0.53 for Mental, Behavioural and Neurodevelopmental disorders (chapter F, 95% CI = 0.31 to 0.93).

## Discussions

### Summary of results

0.5% of death records included an asthma-related cause. When the primary cause of death was asthma-related, ICD-10 code J45 (asthma) rather than J46 (asthma attack) was used 82% of the time. There were higher odds of infection (predominantly pneumonia) being reported as a contributing cause of death when J45 was the primary coded cause, compared to J46 (odds ratio = 7.51, 95% CI = 2.76 to 20.41). When asthma was only a secondary cause, 42% of deaths had primary cause related to diseases of the circulatory system.

### Results in context

The process of completing a death certificate is liable to bias, particularly where there may be very co-morbid patients with chronic diseases. In the 2014 National Review of Asthma Deaths (NRAD), the MCCDs had been predominantly completed by junior doctors and therefore more likely to have inaccuracies than if they were completed by a senior doctor [20]. In an expert panel review of the medical records of 755 people whose underlying causes of death was recorded as asthma, 352 (47%) were excluded during the initial screening as they either did not have asthma or unlikely that asthma caused or contributed to the cause of death [20]. 127 patients were excluded from the review due to insufficient information provided by healthcare providers. 276 patients were passed for more in-depth review, of whom 10% had no evidence of asthma diagnosis at all, and 13% had asthma but did not die from it [21]. This demonstrated the importance of cross-referencing with primary care records for reliable ascertainment of validated asthma deaths.

This bias can influence the accuracy of the final coded record in two different stages: the reporting of the death on the MCCD certificate by the attending physician, and the ICD coding of that certificate by the medical coder. A Japanese 2019 study reviewed 103 asthma ICD-10 coded deaths, and found that 16% were not asthma deaths, and that for 13% the cause could not be ascertained without further investigation [22]. A Swiss study compared the mortality data for in-hospital deaths to their terminal hospital discharge records, and found that for asthma mortality record cases (*n* = 50) there was only 24% agreement as primary cause [23]. Conversely, for the 20 cases with asthma as the principal terminal hospital discharge cause, the agreement to the mortality record was 60%. Unfortunately, we were not able to identify any UK data to directly compare these studies to.

The ontological coding from death certificate to ICD-10 value may also introduce some degree of bias, particularly when conducted by a human-coder rather than automated coding software. A study in the Netherlands compared the results of two independent ICD coders, and found that for respiratory deaths (*n* = 1145) there was agreement to the 4-digit level in 81% of cases, and to the 3-digit level in 84% of cases, but only to the chapter level in 88% of cases [24]. The chapter-level agreement was less than 70% for infectious, endocrine, and skin diseases, but over 95% for neoplasms. Although this study used manual coding, there may also be variation in automated coding between software and versions which affect outputs. An NRS review of the change to Iris in January 2017 from the previous Mortality Medical Data System (MMDS) system, which was an automated system in use from 2000, observed a decrease of 4.8% in the number of deaths allocated to respiratory causes in Scotland. This was mainly due to the switch of deaths from chest infections and aspiration pneumonia to dementia and diseases of the nervous system [25].

The typical standard for asthma death ascertainment from ICD-10 coded data is either J45 or J46 (and lower-level codes under these parents) as the primary cause of death. However, consideration must be paid to patients with a prior misdiagnosis of asthma [26], with an incorrect prior diagnosis of a different respiratory condition [27], with no reported history of asthma diagnosis [21], and with comorbid conditions [21], including infections and overlapping COPD and asthma [20, 21, 27]. As highlighted in our methods, the code J448 includes chronic obstructive asthmatic bronchitis however as it does not differentiate between asthma related and emphysematous related COPD, it was not used as an identification criterion in our study. As such our study may have underestimated any deaths associated with chronic asthmatic bronchitis. More complex rules and exclusions may be required to improve the accuracy of asthma mortality ascertainment, especially if such data were to be used for training disease prediction models.

We investigated changes in practices over the duration of the observed data (2000 to 2017). Variation in practice over time was observed, such as in the use of J45 versus J46 as the UCOD, but we failed to identify any clear trends. Further investigation is required to explore possible causes of temporal changes. Furthermore, the effect of the disruption to the healthcare system resulting from the CoVID-19 pandemic warrants further exploration [28].

The ALHS dataset contains records for a subset of the Scottish population: over half a million patients from 75 general practices in Scotland [19]. In this study population, Scottish people from areas with lower socioeconomic deprivation are slightly over-represented, however the population is otherwise fairly representative.

## Conclusion

One in every 200 deaths in this Scottish dataset, between 2000 and 2017, were coded as being asthma related, as denoted by the inclusion of ICD-10 codes J45 and J46. Infection (predominantly pneumonia) was more commonly reported as a contributing cause of death when J45 was the primary coded cause, compared to J46, which specifically denotes asthma attacks. However, our study highlights the potential impact that bias can have on final cause of death reporting and coding. This is important to consider when creating disease prediction models which utilize retrospective data spanning large population and points in time.

## Data Availability

The ALHS data are held by the National Services Scotland electronic Data Research and Innovation Service (eDRIS) in the National Safe Haven. Restrictions apply to the availability of these data, which were used under license for the current study, and so are not publicly available. Data would be made available from a reasonable request to phs.edris@phs.scot. Code scripts, in the R language, for all components of the data cleaning and analysis are available at https://github.com/hollytibble/asthma_mortality_ICD.

## Abbreviations

ALHS: Asthma Learning Healthcare System study
A&E: Accident and Emergency Department
COPD: Chronic Obstructive Pulmonary Disease
ICD-10: International Classification of Diseases, Version 10
MCCD: Medical Certificate of Cause of Death
MUSE: Multicausal and Unicausal Selection Engine
UCOD: Underlying Cause Of Death
NRAD: National Review of Asthma Deaths
NRS: National Records of Scotland
ONS: the Office for National Statistics
WHO: World Health Organization
